# Supplementary material for: Using sigLASSO to optimize cancer mutation signatures jointly with sampling likelihood
Source: Nat Commun. 2020 Jul 17;11:3575. doi: 10.1038/s41467-020-17388-x (PMC7368050; doi:10.1038/s41467-020-17388-x)
Supplement: Supplementary file 1 — Supplementary Information [file 41467_2020_17388_MOESM1_ESM.pdf]

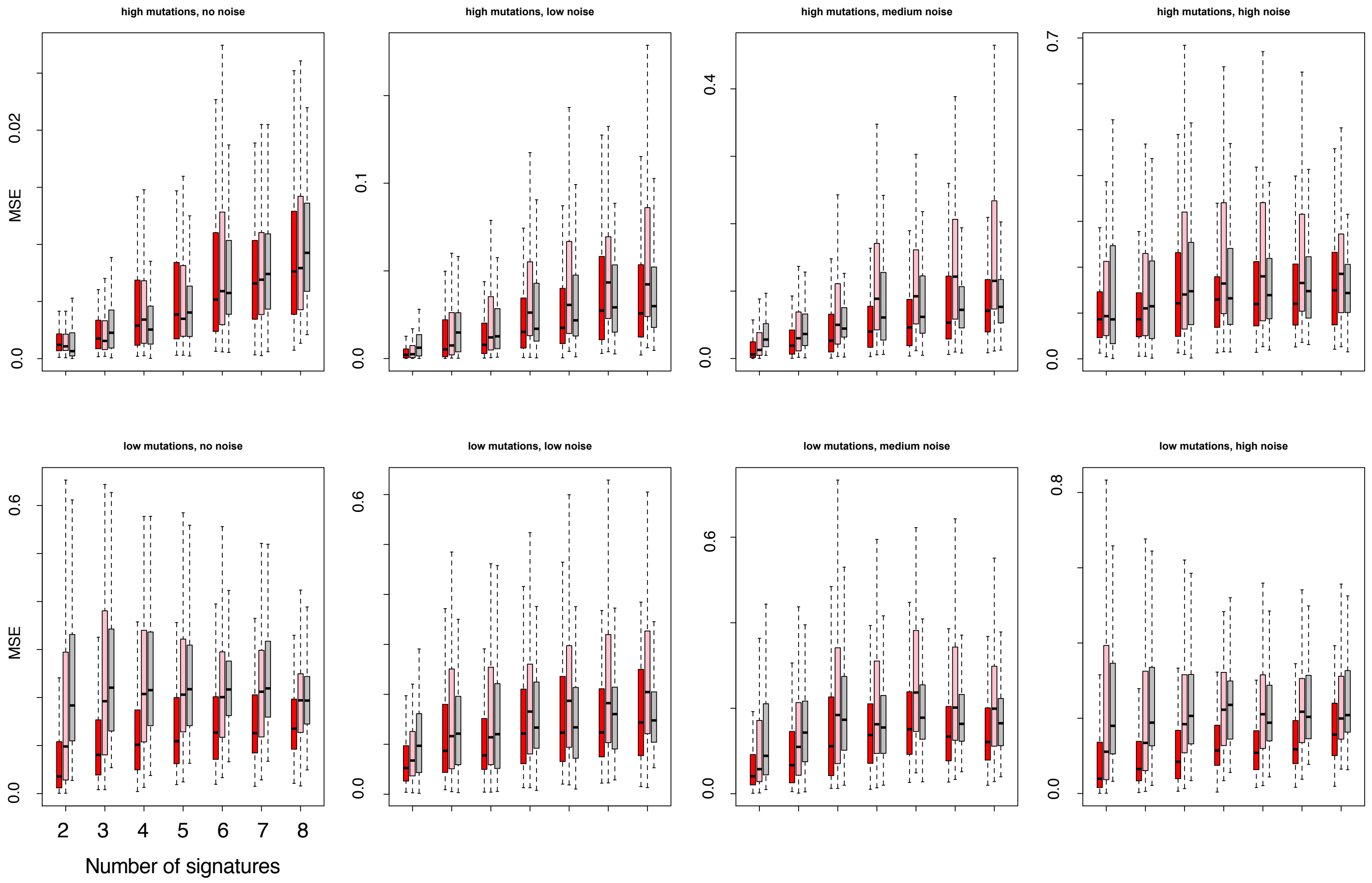

Supplementary Figure 1: Boxplots of MSE on simulated datasets. Red: sigLASSO, pink: sigLASSO (without prior), grey: deconstructSigs.

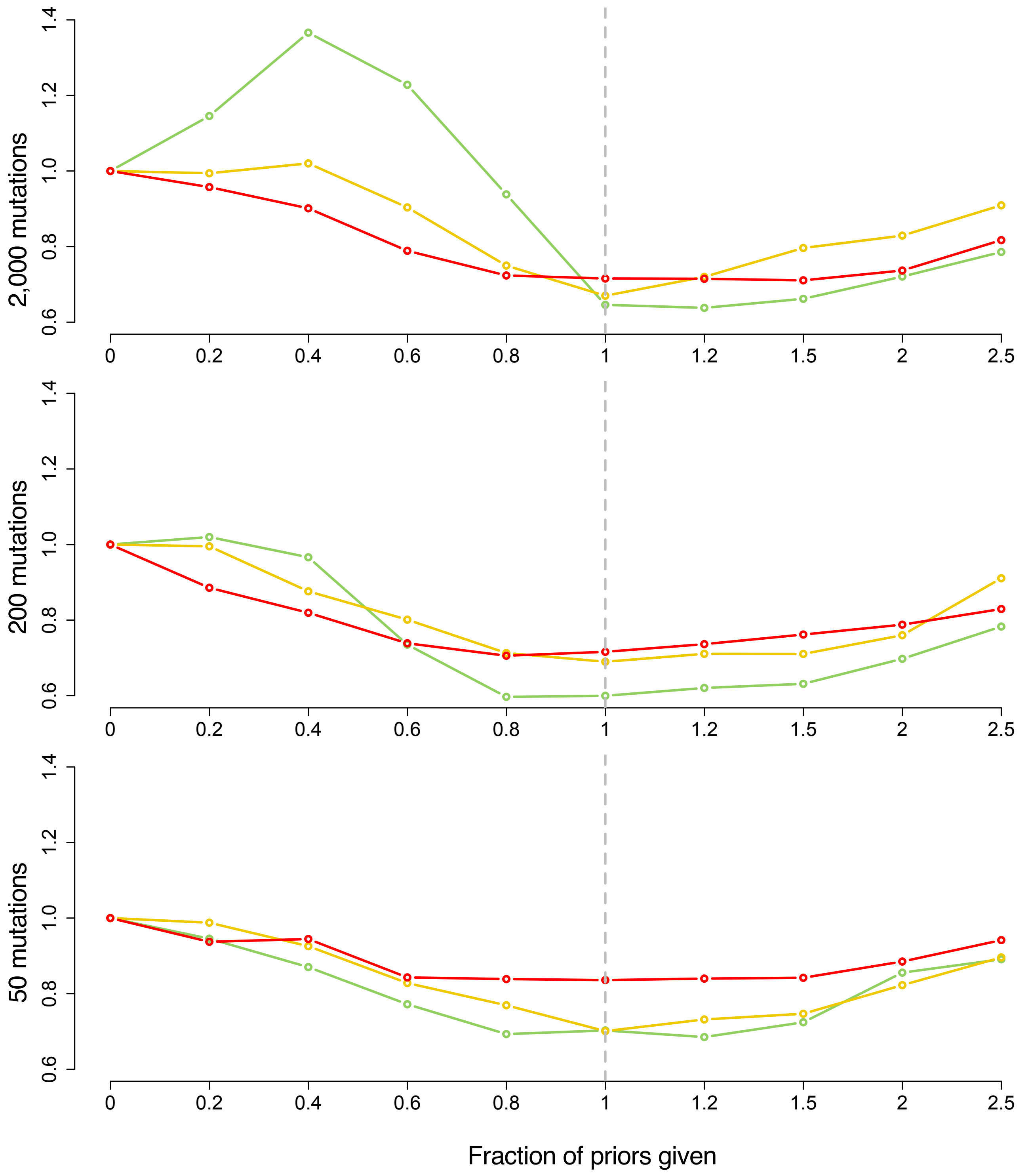

Supplementary Figure 2: Relative MSE on simulated datasets, showing that tuning the penalty weights using prior knowledge improves performance. Penalty weights used: red, 0.5; yellow, 0.1; green, 0.01.

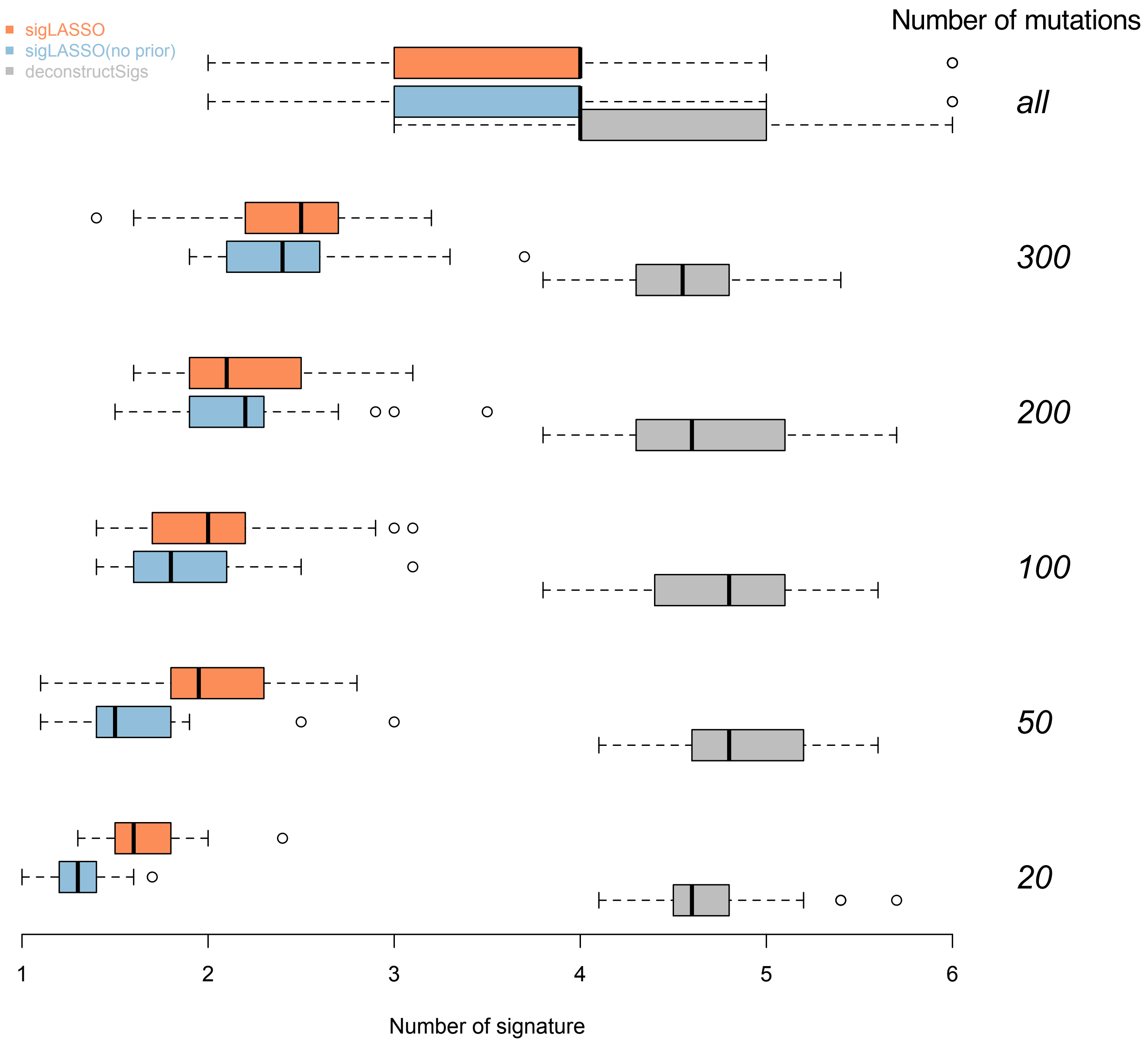

Supplementary Figure 3: Number of active signatures assigned (individually averaged over subsampling) for 30 WGS PRCC samples.

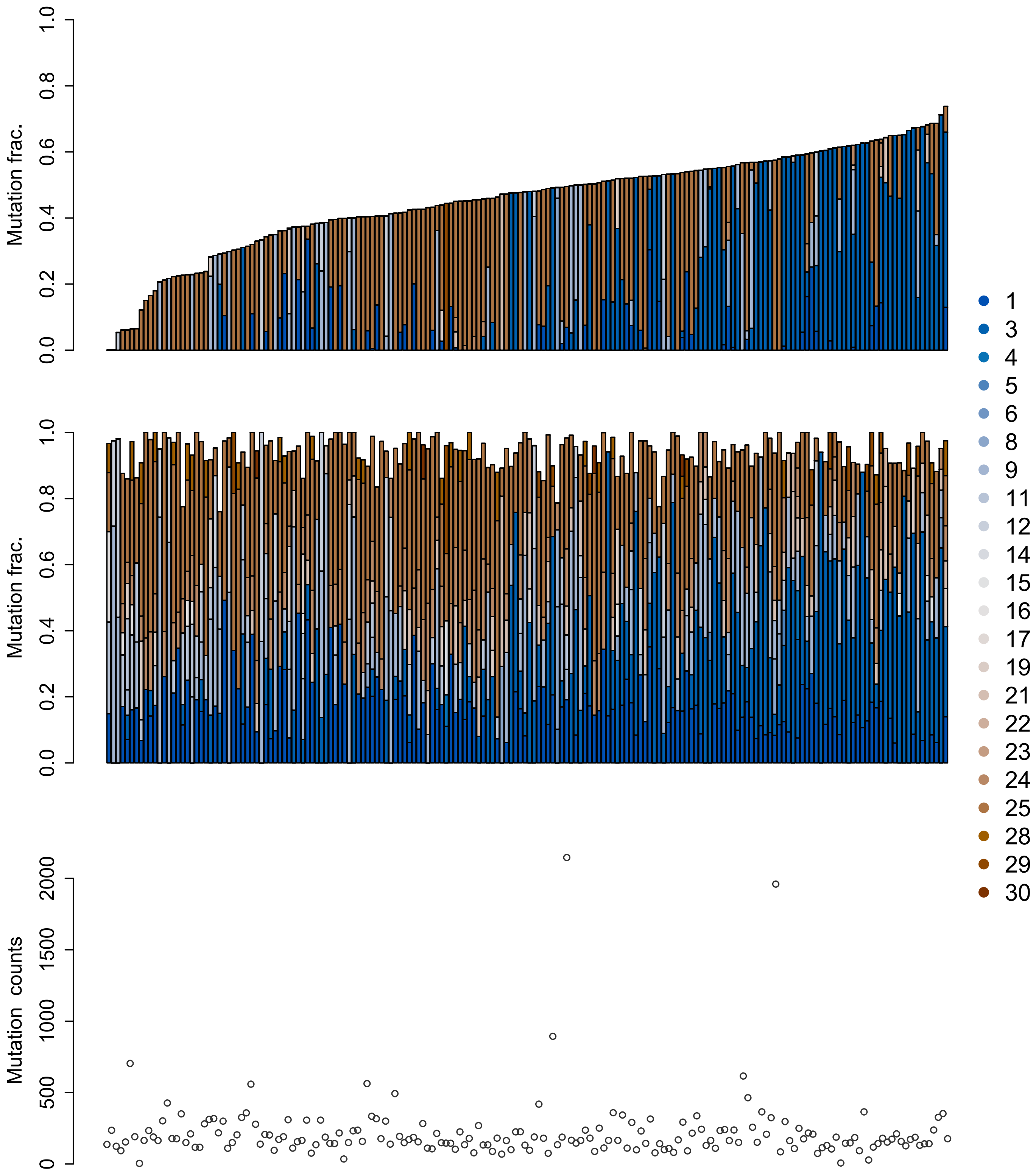

Supplementary Figure 4: Signature assignment for 182 WES ESCA samples. Bar plots show the fractions of mutation signature assignment for each sample using sigLASSO and deconstructSigs, sorted by the fraction of the signature assignment.

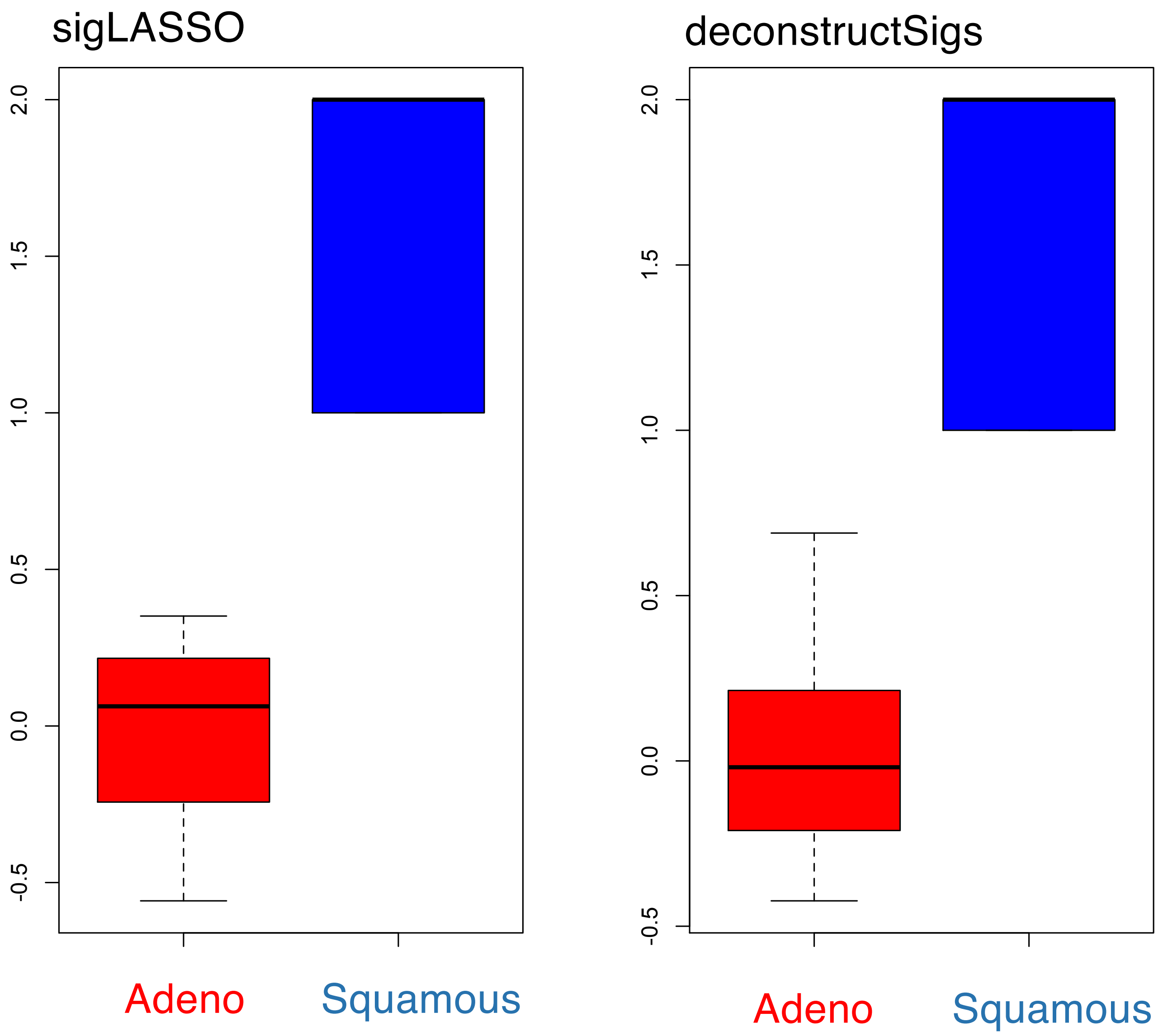

Supplementary Figure 5: Principle component 1(PC1) of signatures assigned for ESCA samples stratified by subtypes. The box indicates the interquartile range (IQR) and whiskers indicate the 1.5x IQR or max/min, which ones are smaller.

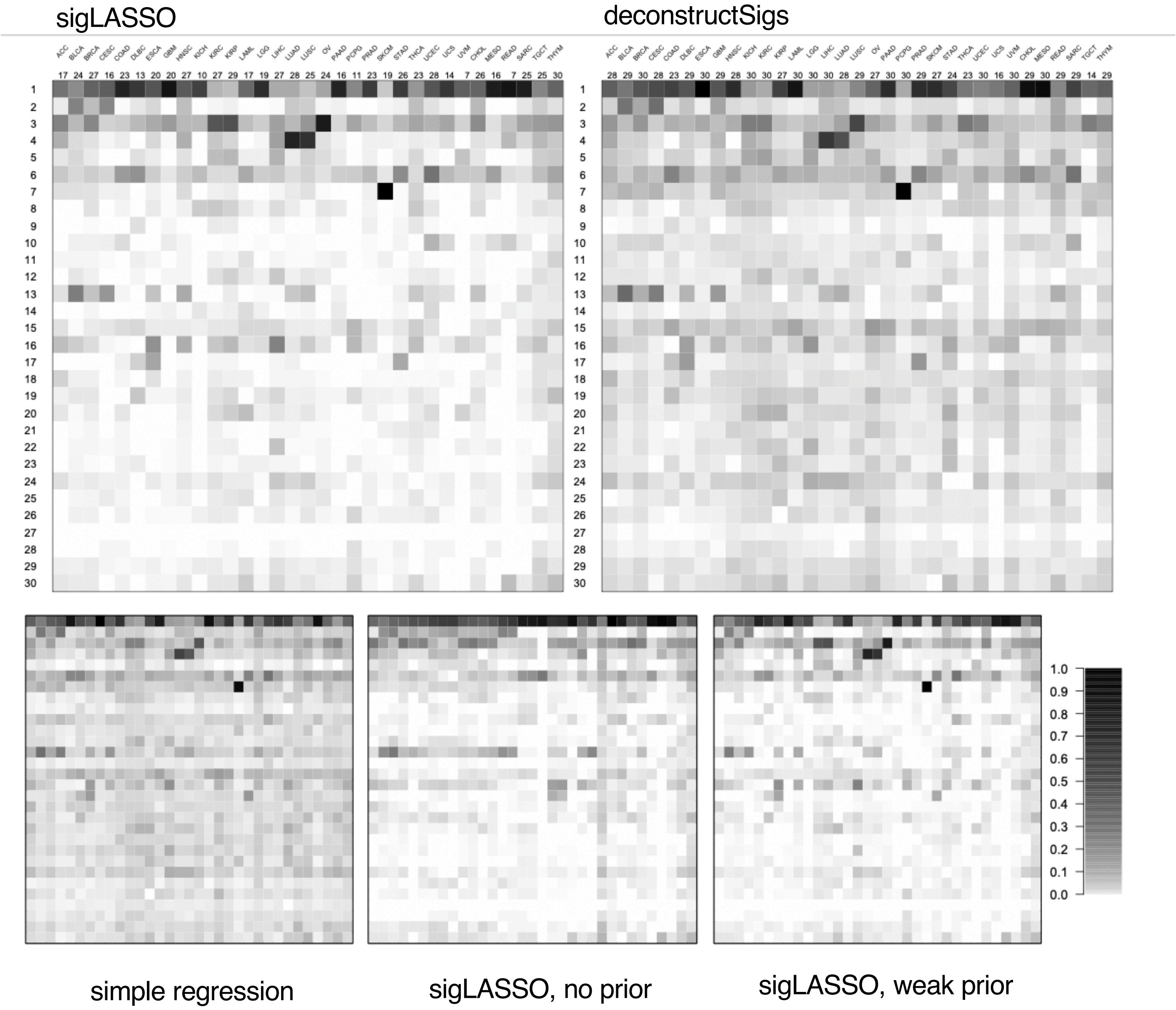

Supplementary Figure 6: Signature assignment in greyscale

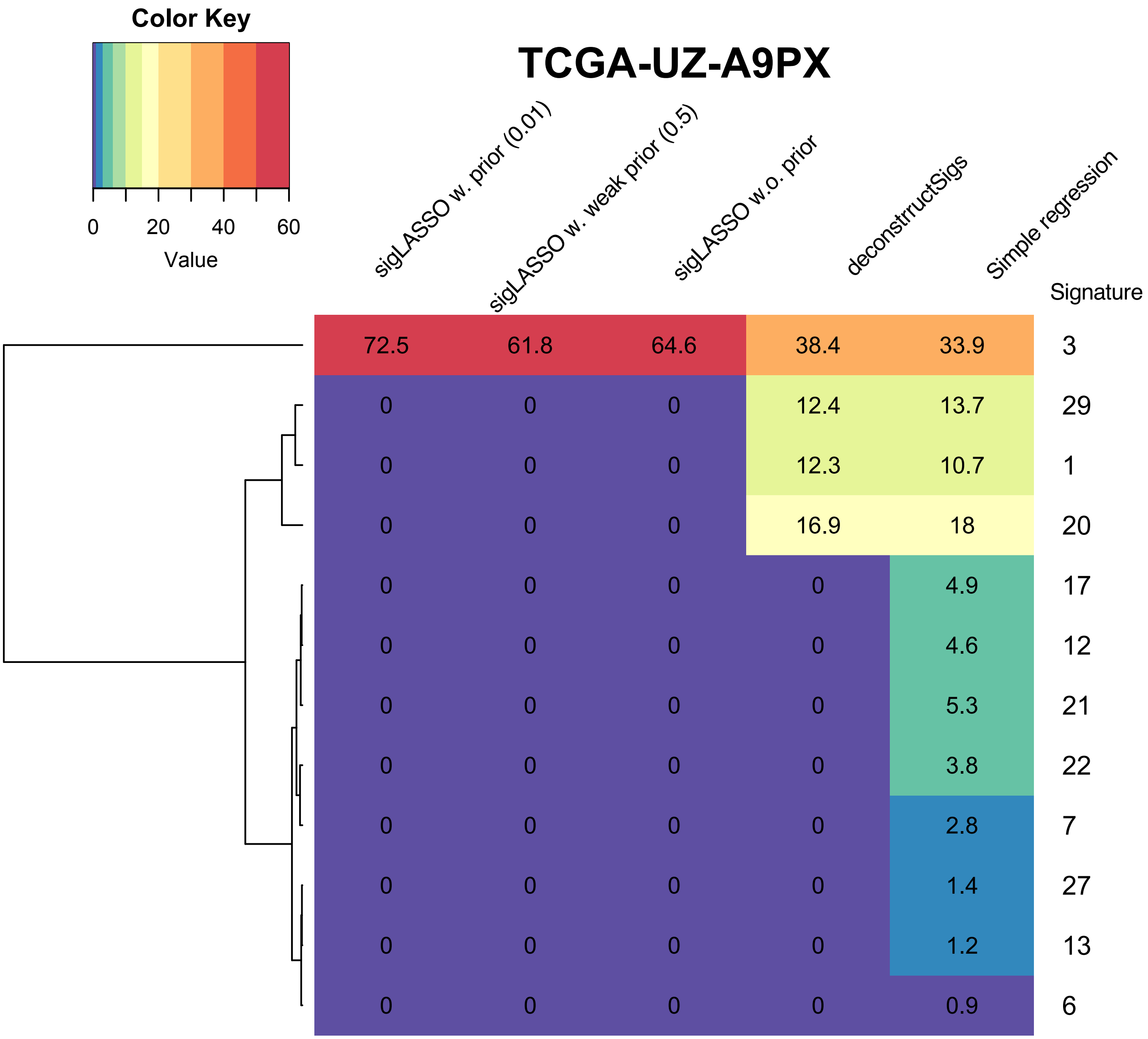

Supplementary Figure 7: A single kidney cancer case (TCGA-UZ-A9PX) illustrating how signature composition (in percentage) changes with different methods and settings. Signatures are hierarchically clustered (as shown in the dendrogram) using the Euclidean distance.

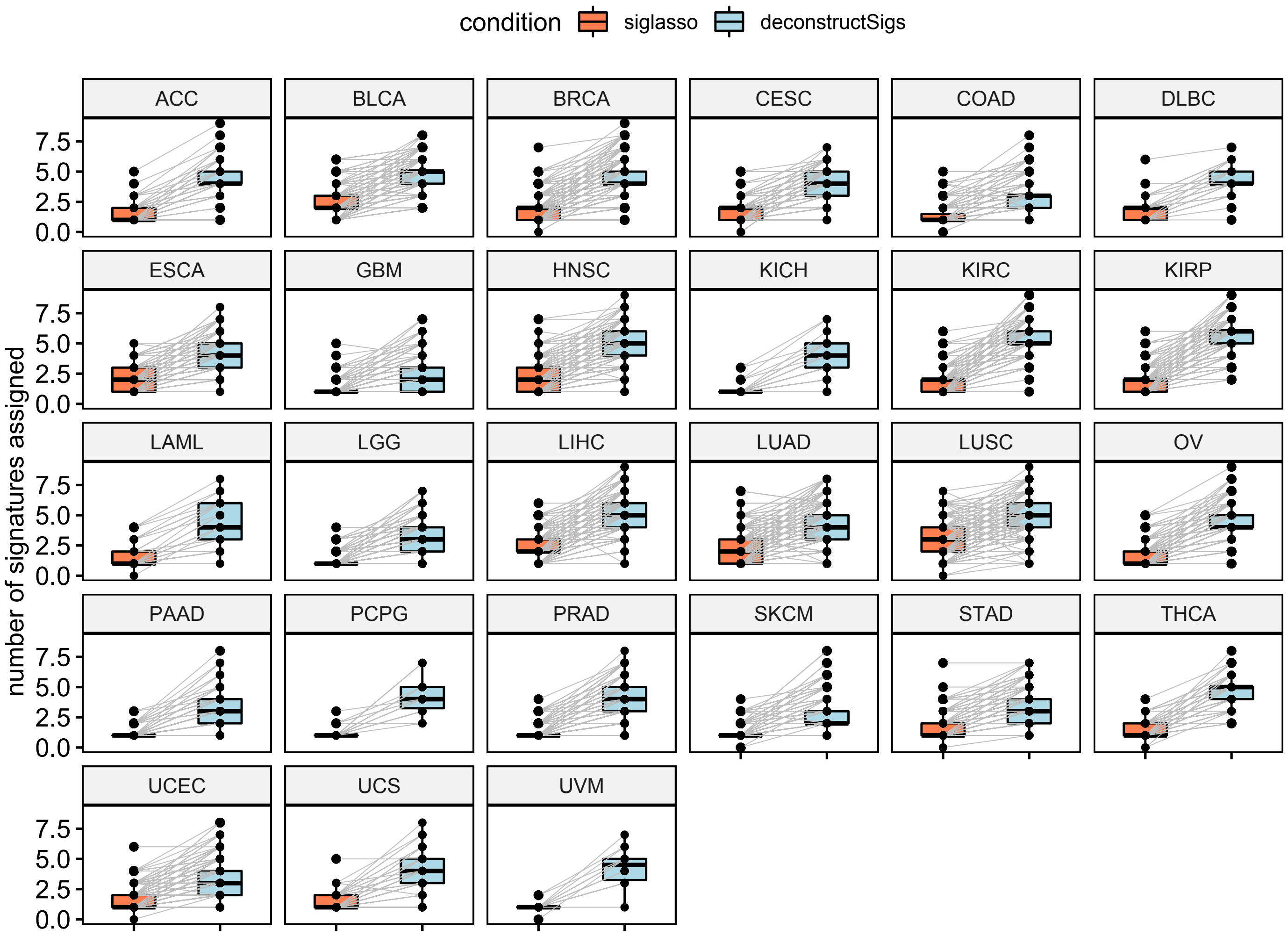

Supplementary Figure 8: A paired boxplot of numbers of signature assigned by two methods, stratified by cancer types.

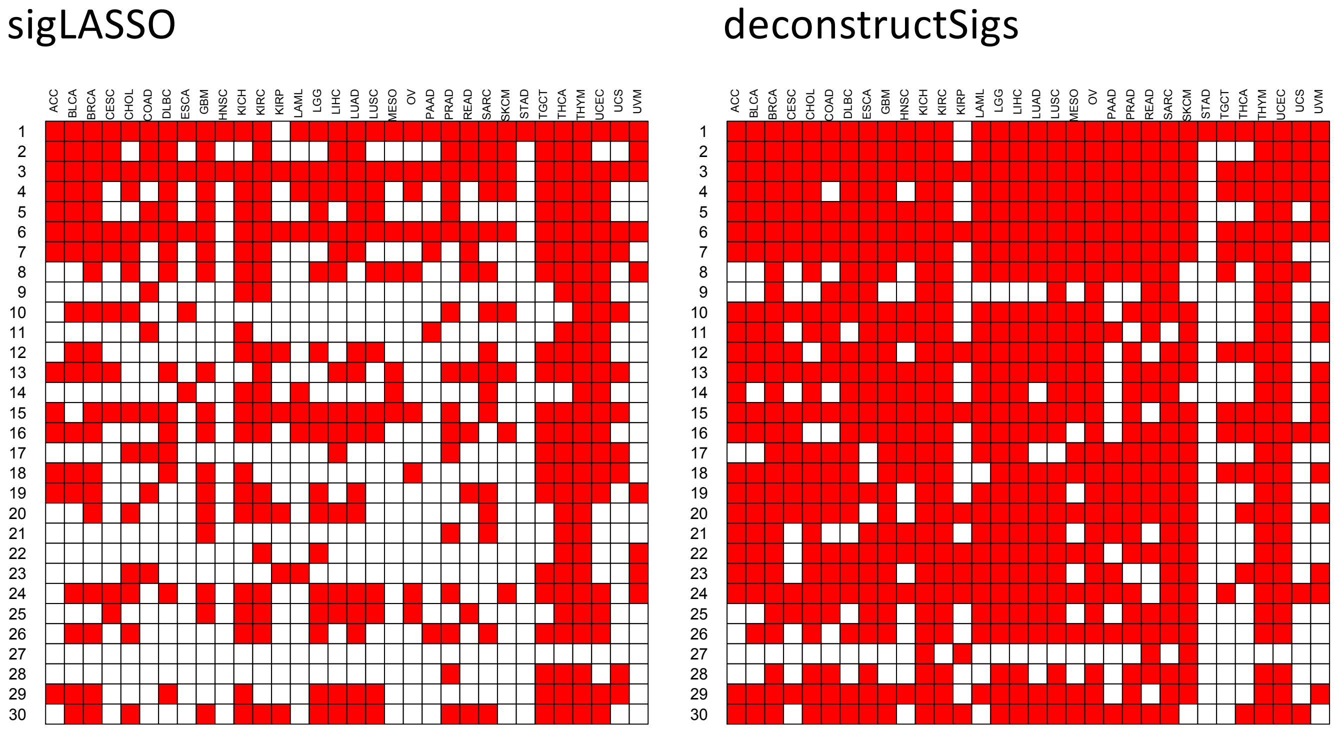

Supplementary Figure 9: changing mutation number cut-off to 50 in WES analysis.

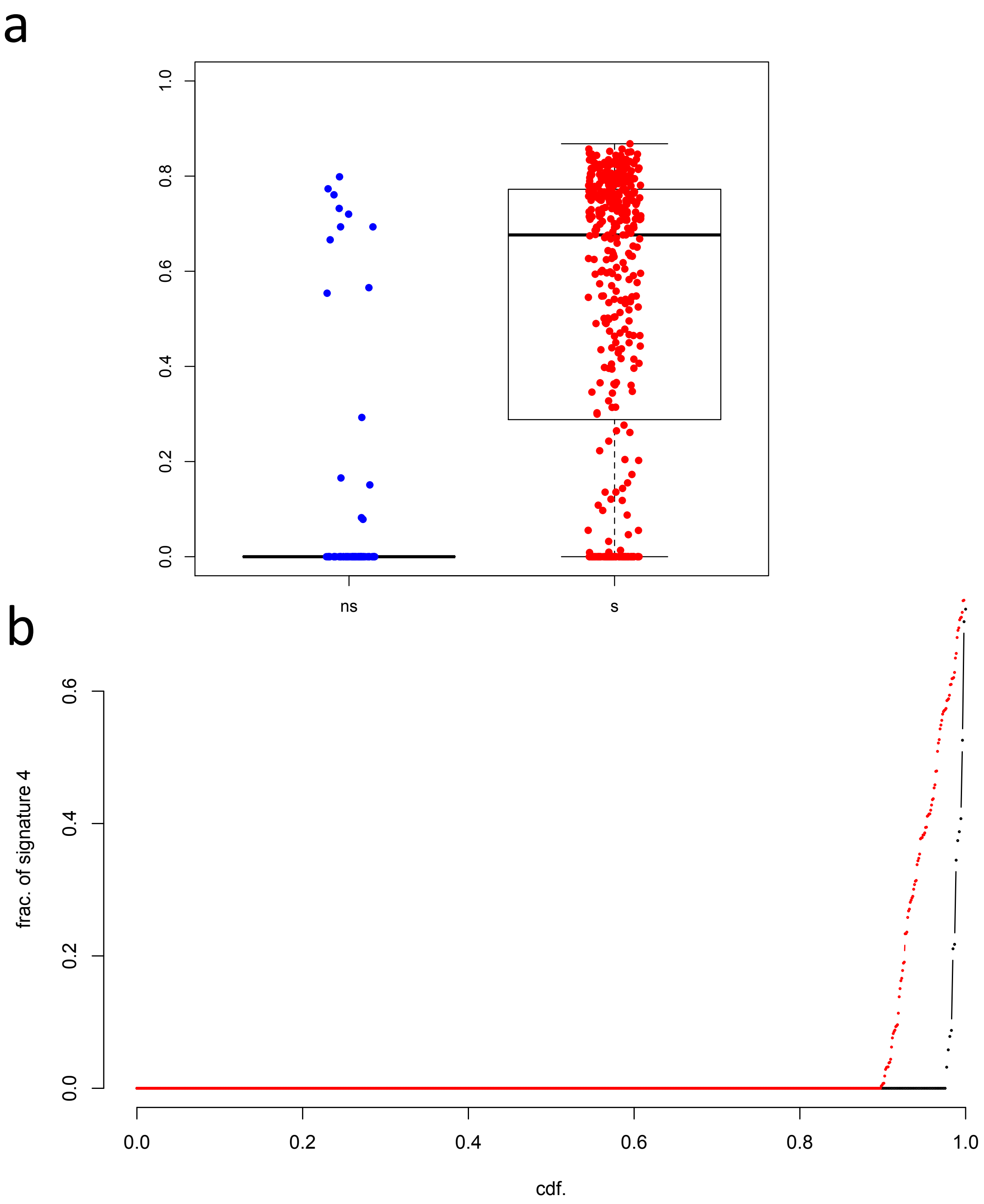

Supplementary Figure 10: **(a)** Boxplot of fractions of signature 4 (“smoking signature”) in LUAD samples. ns: nonsmoking; s: smoking. The box indicates the interquartile range (IQR) and whiskers indicate the 1.5x IQR. **(b)** Cumulative distribution function (cdf) of fractions of signature 4 in non-lung tumor samples (N = 1,500); red: smokers (N = 974); blue: nonsmokers (N = 526).

TCGA.ACC.mutect.81ac2c46-37db-4dcd-923a-061a7ae626a3.DR-10.0.somatic.maf
TCGA.BLCA.mutect.0e239d8f-47b0-4e47-9716-e9ecc87605b9.DR-10.0.somatic.maf
TCGA.BRCA.mutect.995c0111-d90b-4140-bee7-3845436c3b42.DR-10.0.somatic.maf
TCGA.CESC.mutect.5ffa70b1-61b4-43d1-b10a-eda412187c17.DR-10.0.somatic.maf
TCGA.CHOL.mutect.c116f412-e251-4192-9bc5-3ce3cfaaa774.DR-10.0.somatic.maf
TCGA.COAD.mutect.03652df4-6090-4f5a-a2ff-ee28a37f9301.DR-10.0.somatic.maf
TCGA.DLBC.mutect.c3df46a9-85d1-45d4-954a-825313d4a26d.DR-10.0.somatic.maf
TCGA.ESCA.mutect.7f8e1e7c-621c-4dfd-8fad-af07c739dbfc.DR-10.0.somatic.maf
TCGA.GBM.mutect.da904cd3-79d7-4ae3-b6c0-e7127998b3e6.DR-10.0.somatic.maf
TCGA.HNSC.mutect.1aa33f25-3893-4f37-a6a4-361c9785d07e.DR-10.0.somatic.maf
TCGA.KICH.mutect.ddb523ba-29ac-4056-82ca-4147d2e98ddf.DR-10.0.somatic.maf
TCGA.KIRC.mutect.2a8f2c83-8b5e-4987-8dbf-01f7ee24dc26.DR-10.0.somatic.maf
TCGA.KIRP.mutect.1ab98b62-5863-4440-84f9-3c15d476d523.DR-10.0.somatic.maf
TCGA.LAML.mutect.27f42413-6d8f-401f-9d07-d019def8939e.DR-10.0.somatic.maf
TCGA.LGG.mutect.1e0694ca-fcde-41d3-9ae3-47cfaf527f25.DR-10.0.somatic.maf
TCGA.LIHC.mutect.a630f0a0-39b3-4aab-8181-89c1dde8d3e2.DR-10.0.somatic.maf
TCGA.LUAD.mutect.0458c57f-316c-4a7c-9294-ccd11c97c2f9.DR-10.0.somatic.maf
TCGA.LUSC.mutect.95258183-63ea-4c97-ae29-1bae9ed06334.DR-10.0.somatic.maf
TCGA.MESO.mutect.88b38a05-e46a-49e1-9c4d-e098709256b1.DR-10.0.somatic.maf
TCGA.OV.mutect.b22b85eb-2ca8-4c9f-a1cd-b77caab999bd.DR-10.0.somatic.maf
TCGA.PAAD.mutect.fea333b5-78e0-43c8-bf76-4c78dd3fac92.DR-10.0.somatic.maf
TCGA.PCPG.mutect.64e23e2f-ec04-4f6b-82b3-375e2d49804b.DR-10.0.somatic.maf
TCGA.PRAD.mutect.deca36be-bf05-441a-b2e4-394228f23fbe.DR-10.0.somatic.maf
TCGA.READ.mutect.faa5f62a-2731-4867-a264-0e85b7074e87.DR-10.0.somatic.maf
TCGA.SARC.mutect.cc207fe8-ee0a-4b65-82cb-c8197d264126.DR-10.0.somatic.maf
TCGA.SKCM.mutect.4b7a5729-b83e-4837-9b61-a6002dce1c0a.DR-10.0.somatic.maf
TCGA.STAD.mutect.c06465a3-50e7-46f7-b2dd-7bd654ca206b.DR-10.0.somatic.maf
TCGA.TGCT.mutect.6f6a4290-b6be-49f5-be45-97d742957a9e.DR-10.0.somatic.maf
TCGA.THCA.mutect.13999735-2e70-439f-a6d9-45d831ba1a1a.DR-10.0.somatic.maf
TCGA.THYM.mutect.91ddbf37-6429-4338-89df-2d246a8e2d00.DR-10.0.somatic.maf
TCGA.UCEC.mutect.d3fa70be-520a-420e-bb6d-651aeee5cb50.DR-10.0.somatic.maf
TCGA.UCS.mutect.02747363-f04a-4ba6-a079-fe4f87853788.DR-10.0.somatic.maf
TCGA.UVM.mutect.6c7b01bc-b068-4e01-8b4d-0362f5959f65.DR-10.0.somatic.maf

Supplementary Table 1: TCGA MAF files used in this study
